# ID 59 - Análise Econômica e Sustentável do Reprocessamento de Máscaras N95/PFF2

**DOI:** 10.5327/2237-9622.2025.v34s1.59

**Published:** 2025-11-25

**Authors:** Wanderson Eduardo Gomes de Souza Coelho, Taminato Wanderson Eduardo Coelho, Laís Maria de Campos, Deyvid Fernando Mattei, Daiane Pereira Carneiro, Ana Carolina Goulardins Almeida, Marcos Viana Moura, Hugo Fernandes, Monica Taminato

**Keywords:** covid-19, máscaras N95, descontaminação, peróxido de hidrogênio, custos e análise de custo, sustentabilidade

## Abstract

**Introdução:**

A pandemia da covid-19 impactou significativamente a economia global, além dos milhões de casos e mortes registrados até janeiro de 2024 estudos demonstram os impactos negativos no meio ambiente e na economia. Durante o período pandêmico, os profissionais de saúde foram especialmente afetados, com altos riscos de infecção e morte, devido à escassez de equipamentos de proteção individual, como máscaras N95/PFF2. Diante desse cenário, medidas de otimização foram necessárias, incluindo, em alguns países, a descontaminação para reutilização, uma vez que a demanda superou a oferta de respiradores, aumentando também a quantidade de resíduos gerados. Assim, considerando a demanda crescente por cuidados de saúde, a possibilidade de futuras situações de contingência, os impactos ambientais dos resíduos gerados e o aumento da extração de matéria-prima em um cenário de restrições orçamentárias e limitações de recursos, este estudo tem como objetivo avaliar a viabilidade econômica e impacto ambiental da descontaminação de máscaras N95/PFF2 por peroxido de hidrogênio, contribuindo para uma gestão eficiente, transparente, equitativa e racional dos recursos disponíveis.

**Método:**

Estudo de avaliação econômica realizado em junho de 2023 em um hospital público no Brasil, com 150 leitos, que utilizou a metodologia de microcusteio, com base na modelagem de atendimento a pacientes com covid-19. A metodologia proposta por Sionk Swan Tan foi utilizada para estimar os custos, seguindo etapas que incluíram definição da perspectiva de análise; definição da unidade de análise; identificação de itens de custo; mensuração dos itens de custo e valoração dos itens de custo. A checklist CHEER-2 foi utilizada para elaboração do estudo.

**Resultados:**

Entre abril de 2020 e março de 2023, estimou-se o consumo de 67.490 máscaras N95/PFF2 para o atendimento de pacientes com covid-19. O estudo indicou que o custo total para aquisição e descarte adequado dessas máscaras seria de R$ 237.753,77. No entanto, com o método de reprocessamento, esse custo poderia ser reduzido para R$ 94.945,71, gerando uma economia de 60%. Além disso, o reprocessamento teria o potencial de diminuir a geração de resíduos em 81,4%.

**Conclusão:**

O reprocessamento de máscaras N95/PFF2 demonstrou ser uma estratégia economicamente viável e ambientalmente sustentável. A economia gerada aliada à redução de resíduos evidencia os benefícios do reprocessamento, tanto em termos financeiros quanto de preservação ambiental. No âmbito ambiental, os dados reforçam a preocupação com o aumento da poluição plástica, causada pelo descarte inadequado de equipamentos de proteção individual (EPIs). Essa realidade coloca em destaque a importância de buscar soluções sustentáveis, como o reprocessamento de máscaras, que reduz significativamente o impacto ambiental. Embora a literatura já confirme a segurança do reprocessamento de máscaras, a legislação brasileira atual proíbe essa prática, indicando a necessidade de uma revisão das políticas sanitárias no País. Uma abordagem baseada em evidências, poderia embasar mudanças regulatórias que favoreçam o uso racional e eficiente de recursos, não apenas financeiros, mas também materiais e naturais. A implementação de diretrizes de avaliação econômica em saúde pode garantir o acesso equitativo a tecnologias eficazes e seguras, otimizando o uso dos recursos disponíveis e promovendo uma gestão de saúde mais eficiente e sustentável no Brasil.

